# Praxis der perioperativen Prävention von Phantomschmerz: eine deutschlandweite Umfrage

**DOI:** 10.1007/s00101-022-01188-7

**Published:** 2022-08-29

**Authors:** Jan D. Wandrey, Michael Schäfer, Joachim Erlenwein, Sascha Tafelski

**Affiliations:** 1 Klinik für Anästhesiologie mit Schwerpunkt operative Intensivmedizin, Campus Charité-Mitte und Campus Virchow-Klinikum, Charité – Universitätsmedizin Berlin, Gliedkörperschaft der Freien Universität Berlin, der Humboldt Universität zu Berlin und des Berlin Institute of Health, Charitéplatz 1, 10117 Berlin, Deutschland; 2 Wissenschaftlicher Arbeitskreis Schmerzmedizin der Deutschen Gesellschaft für Anästhesiologie und Intensivmedizin (DGAI), Nürnberg, Deutschland; 3 Universitätsmedizin Göttingen, Klinik für Anästhesiologie, Georg-August-Universität, Robert-Koch-Straße 40, 37075 Göttingen, Deutschland

**Keywords:** Neuropathischer Schmerz, Phantomschmerz, Postoperative Schmerzen, Prävention und Kontrolle, Umfragen und Fragebögen, Neuropathic pain, Phantom limb pain, Postoperative pain, Prevention and control, Surveys and questionnaires

## Abstract

**Hintergrund:**

Phantomschmerzen haben eine hohe Prävalenz nach Majoramputationen und sind mit einer zusätzlichen Einschränkung der Lebensqualität verbunden. Perioperative Behandlungsstrategien könnten zur Prävention von Phantomschmerzen beitragen. Diese Studie soll die aktuelle Praxis des perioperativen anästhesiologischen Schmerzmanagements bei Majoramputation darstellen, eine Einschätzung des Optimierungspotenzials und eine Barriereanalyse für die Versorgung dieser Patientenpopulation erarbeiten.

**Material und Methoden:**

In einer Onlineumfrage aus dem Wissenschaftlichen Arbeitskreis Schmerzmedizin der Deutschen Gesellschaft für Anästhesiologie e. V. (DGAI) wurden alle Fachärztinnen und Fachärzte für Anästhesiologie der Gesellschaft um Teilnahme gebeten und anonymisiert befragt.

**Ergebnisse:**

Insgesamt 402 Antworten zeigten, dass aktuell meist eine Allgemeinanästhesie (85 %), ein Verfahren der Leitungs- oder Plexusanästhesie (63 %) oder eine rückenmarknahe Anästhesie (49 %) in unterschiedlichen Kombinationen durchgeführt wurden. Des Weiteren gaben 72 % der Antwortenden an, postoperativ i.v.-Opioide zu nutzen, wobei 57 % eine patientenkontrollierte Analgesie (PCA) verwendeten. Demgegenüber wurden beim Einsatz präoperativer Regionalverfahren (74 %) und präoperativer Gabapentinoide (67 %) Ansätze zur Behandlungsoptimierung gesehen. Insbesondere organisatorische wie auch patientenimmanente Faktoren wurden als Barrieren bei der Versorgung benannt.

**Diskussion:**

Die Umfrage bildet die aktuelle Praxis des perioperativen Schmerzmanagements bei Majoramputationen ab. Es deutet sich an, dass Bedarf für eine bereits präoperativ eingebundene, schmerzmedizinische Behandlung besteht. Vor dem Hintergrund der eingeschränkten Evidenz von aktuellen Therapieempfehlungen lassen sich aus der dargestellten Versorgungspraxis Fragestellungen für weitere Studien ableiten.

## Abstract (en)

**Background:**

The prevalence of phantom limb pain after major amputation remains high and affected patients suffer from relevant impairments in the quality of life. Perioperative treatment strategies may prevent phantom limb pain. This study aims to assess the state of the perioperative anesthesiological pain management for major amputations. Furthermore, it analyzes potentials for optimization and barriers towards a better treatment of patients with amputations.

**Material and methods:**

This online survey was distributed by the German Society of Anesthesiology and Intensive Care Medicine (DGAI) mailing list of anesthesiological consultants. It was approved by the Charité ethics board.

**Results:**

Overall, 402 persons participated in this survey. Mostly, general anesthesia (85%), regional anesthesia (63%) and neuraxial anesthesia (49%) were performed in different combinations. Furthermore, 72% of participants reported using i.v. opioids postoperatively, mostly applied via patient-controlled analgesia (PCA). In contrast, preoperative regional anesthesia (74%) and gabapentinoids (67%) were seen as potential methods to improve treatment; however, barrier analysis revealed that treatment options are limited especially by organizational aspects and intrinsic patient factors.

**Conclusion:**

This survey describes the current practice of phantom limb pain prevention in Germany. It shows an unmet need for specialized perioperative pain treatment. As the evidence regarding treatment recommendations is limited, further research questions can be deduced from this study.

## Hintergrund und Fragestellung

Die Therapie postoperativer Schmerzen sowie die Prävention einer Chronifizierung sind für viele Patientinnen und Patienten unzureichend, führen zu Einschränkungen der Lebensqualität und stellen ein globales Gesundheitsproblem dar [5, 21, 24]. Somit ist bereits die Reduktion des Risikos einer solchen Chronifizierung eines der zentralen Anliegen in der perioperativen, schmerztherapeutischen klinischen Praxis und Forschung. Diese Aspekte sind gerade auch im Kontext von Majoramputationen von Relevanz, da sensorische Phänomene wie Phantomschmerzen oft auftreten und häufig chronifizieren. In Deutschland wurden im Jahr 2014 insgesamt 57.637 Amputationen durchgeführt, von denen knapp 29 % Majoramputation waren [17]. Die Prävalenz von Phantomschmerzen bei Amputierten liegt weltweit bei ca. 64 %, wobei die Daten je nach Studie sehr schwanken [18]. Dabei führt Phantomschmerz zu einer relevanten Einschränkung der Lebensqualität [4]. Bis heute ist die Evidenz zu Therapieoptionen aufgrund fehlender Studien mit relevanten Fallzahlen und randomisiertem, kontrolliertem Design sehr gering [7, 27, 31]. Auch in der klinischen Praxis werden die Beschwerden oft nur unbefriedigend behandelt, weshalb der Prävention der Erkrankung in der perioperativen Phase ein besonderer Stellenwert zukommt [7, 27, 31]. Eine frühzeitige Einbeziehung schmerzmedizinischer Expertise scheint sinnvoll zu sein, da zahlreiche Studien eine Assoziation von Präamputationsschmerz und postoperativem Akutschmerz mit der Entstehung von Phantomschmerzen zeigen konnten [18, 31]. In einer Arbeit von Gerbershagen et al. konnte gezeigt werden, dass häufig die Schmerzen nach kleineren Operationen am ersten postoperativen Tag die der größeren Operationen überwogen. So lag beispielsweise die transfemorale Oberschenkelamputation lediglich auf Platz 115 von 179 der schmerzhaftesten Operationen [11]. Eine mögliche Interpretation liegt darin, dass bei größeren Operationen aufgrund stärkerer Awareness suffiziente Schmerztherapien eingesetzt werden. Solche präventiven Ansätze könnten auch aus pathophysiologischen Überlegungen Sinn ergeben, in denen Phantomschmerzen als eine Form von maladaptiver ZNS-Plastizität oder durch Mechanismen im peripheren Nervensystem beschrieben werden [4, 9]. Bislang sind keine spezifischen und validen Konzepte für das schnittstellenübergreifende Schmerzmanagement vor und nach Amputationen etabliert. Ebenso fehlt es oft an entsprechenden spezifischen innerklinischen und ambulanten Strukturen für die Behandlung von Patientinnen und Patienten mit Amputationen. Es bedarf also aufgrund der hohen Relevanz für Betroffene der Entwicklung entsprechender spezifischer Konzepte für die Amputationsbehandlung. Wünschenswert wäre vor diesem Hintergrund, die Prävention von Phantomschmerzen in den Fokus der Weiterentwicklung dieser perioperativen Behandlungsstrategien zu rücken. Die im Frühjahr veröffentlichte S3-Leitlinie zur Akutschmerztherapie betont den Stellenwert dieses Themas und erörtert es mit Methoden der evidenzbasieren Medizin [31]. Maßnahmen zur Prävention von Phantomschmerz sollten eine frühzeitige Integration von pharmakologischen und nichtpharmakologischen Interventionen durch ein interdisziplinäres Team beinhalten, angelehnt an multimodale Therapiekonzepte zur Behandlung chronischer Schmerzen [7]. Für die Weiterentwicklung von Konzepten ist es Voraussetzung, neben der Abbildung der gegenwärtigen Behandlungspraxis Barrieren zu erkennen, die einer Implementierung von evidenzbasierten Empfehlungen entgegenstehen könnten.

Die vorliegende Umfrage dient vor diesem Hintergrund der Erhebung der aktuellen Praxis des perioperativen Schmerzmanagements von Patientinnen und Patienten mit Majoramputationen, soll Möglichkeiten einer optimierten Behandlung aus Sicht von anästhesiologischen Fachärztinnen und Fachärzten abbilden und mögliche Barrieren der Implementierung identifizieren.

## Studiendesign und Untersuchungsmethoden

Die Studie wurde als Onlinebefragung des Wissenschaftlichen Arbeitskreises Schmerzmedizin der Deutschen Gesellschaft für Anästhesiologie und Intensivmedizin e. V. (DGAI) entworfen. Die Aufforderung zur Teilnahme und ein entsprechender Link wurden an die fachärztlichen Mitglieder der DGAI (insgesamt bis zu 12.653 Fachärztinnen und Fachärzte) versendet. Die Umfrage war über 3 Monate vom 25.02.2021 bis zum 24.05.2021 zur Beantwortung freigeschaltet. Technisch erfolgte die Umfrage mit dem Umfragetool REDCap (Research Electronic Data Capture, Version 11.01.2011 – © 2021 Vanderbilt University, USA), das von der Charité-Universitätsmedizin als Mitglied im REDCap-Konsortium gehostet wurde [12]. Die Umfrage wurde explizit anonym gestaltet und auf die Erhebung personenbezogener oder personenbeziehbarer Daten verzichtet.

Der Fragebogen wurde von den Autoren entworfen und mehrfach von fachlich versierten Kolleginnen und Kollegen getestet und inhaltlich und sprachlich angepasst. Er enthielt zunächst allgemeine Fragen zur antwortenden Person und der Versorgungseinrichtung, in der diese tätig ist, ohne dabei Rückschlüsse auf die Person zuzulassen. Im Anschluss erhielten die Antwortenden eine Fallvignette mit der Bitte um Charakterisierung der typischen perioperativen Versorgung:

Wir würden uns für Ihren typischen Ablauf der Patientenversorgung für folgenden hypothetischen Patienten interessieren:

Ein 45-jähriger Patient wird zur Planung einer transfemoralen Oberschenkelamputation im mittleren Drittel bei Chondrosarkom im Kniegelenk vorgestellt. In der präoperativen Evaluation berichtet der Patient, aufgrund der progredienten Knieschmerzen bislang Ibuprofen, 600 mg, bei Bedarf in Eigenmedikation eingenommen zu haben. Weitere Auffälligkeiten hinsichtlich Nebenerkrankungen, Medikamenten- oder Drogeneinnahme bestehen nicht.

Anschließend wurden die Teilnehmerinnen und Teilnehmer gebeten, diese Fragestellung aus ihrer Sicht nochmals zu beantworten und eine zukünftige, optimierte Behandlungsstrategie zu charakterisieren. Daraufhin folgten Fragen zu Gründen der Abweichung von dieser subjektiv optimalen perioperativen Schmerztherapie von der aktuell tatsächlich durchgeführten Schmerztherapie.

Das Ausfüllen der Umfrage war freiwillig, und im Fragebogen waren Antworten mit „weiß nicht“ oder „keine Angabe“ sowie Freitextkommentare möglich. Die Beantwortung des Fragebogens dauerte ca. 10 min. Die Analyse der Vollständigkeit der Antworten erfolgte über REDCap. Auch unvollständig beantwortete Fragebogen wurden in die Analyse eingeschlossen. Auf Grundlage der Online-Datenbank erfolgte die Auswertung der Umfrage somit auf der Basis aller vorliegenden Antworten. Für die jeweiligen Fragen wurden die absolute Zahl der Antworten sowie der prozentuale Anteil an allen Antworten dieser Frage ausgegeben. Die deskriptive Auswertung erfolgte mit den Analysetools von REDCap, während die statistische Auswertung mit SPSS 26 durchgeführt wurde. Kategoriale Größen wurden mittels Häufigkeiten und in Prozent angegeben. Für den Vergleich der Antworten zwischen aktueller und optimaler Therapie wurde zur Prüfung auf statistische Signifikanz der Wilcoxon-Rangsummentest verwendet. Für alle statistischen Analysen wurde ein α‑Level < 0,05 % gewählt. Die Freitextkommentare wurden geclustert und semiquantitativ ausgewertet. Die Prozentzahlen der quantitativen Ergebnisse beziehen sich auf die jeweilige Gesamtzahl der Antworten auf diese Frage und wurden auf ganze Zahlen gerundet. Die Durchführung der Studie und die Erstellung des Manuskriptes folgten in Anlehnung an die Empfehlungen der CHERRIES-Checkliste [8]. Die Studie erhielt die Zustimmung der Ethikkommission der Charité (EA1/034/20).

## Ergebnisse

Insgesamt beantworteten 402 Ärztinnen und Ärzte die Umfrage, 81 % (324/402) der Antworten waren vollständig. Persönliche Rückmeldungen zur Umfrage an die Autoren und Stichproben ergaben, dass nicht für alle Fachärztinnen und Fachärzte eine (gültige) E‑Mail-Adresse vorlag und nicht an alle bekannten Adressaten der DGAI e. V. eine E‑Mail durchgestellt wurde und möglicherweise Filtertechnologien der Einrichtungen die Nachrichten blockierten.

Die knappe Mehrheit der antwortenden Fachärztinnen und Fachärzte waren Oberärztinnen und Oberärzte sowie Chefärztinnen und Chefärzte (56 %, *n* = 213), und die Teilnehmenden arbeiteten relativ ausgeglichen in Krankenhäusern der Schwerpunkt‑/Zentralversorgung (30 %, *n* = 117), der Regel- und Grundversorgung (26 %, *n* = 102) sowie in Universitätskliniken (24 %, *n* = 92). Mehr als 59 % der Antwortenden gaben für ihr Haus an, dass dort pro Jahr weniger als 50 Majoramputationen durchgeführt würden, wobei 21 % (*n* = 76) keine spezifischen Angaben gemacht haben. Die überwiegende Mehrheit gab an, dass ihre Klinik über SOP zur perioperativen Versorgung verfügte, allerdings lediglich in 33 % der Fälle mit spezifischen Inhalten zur schmerztherapeutischen Versorgung von Majoramputationen (Tab. 1).

**Table 1:** 

Teilnehmende Versorgungseinrichtungen	Häufigkeit % (*n*)
*In welcher Versorgungseinrichtung arbeiten Sie?*	
Universitätsklinik	24 % (92)
Maximalversorgung	12 % (46)
Schwerpunkt‑/Zentralversorgung	30 % (117)
Regel‑/Grundversorgung	26 % (102)
Ambulantes Operationszentrum	2 % (6)
Sonstiges	5 % (19)
Keine Angabe	2 % (6)
*Hat Ihre Versorgungseinrichtung einen der folgenden Schwerpunkte?*	
Zertifiziertes Traumazentrum	63 % (241)
Zentrum Amputationsmedizin	2 % (9)
Keinen dieser Schwerpunkte	31 % (117)
Sonstiges	3 % (13)
*Welche Zertifizierungsstufe hat Ihr Traumazentrum?*	
Überregional	53 % (125)
Regional	34 % (80)
Lokal	13 % (31)
*Durchschnittliche Anzahl von Majoramputation pro Jahr*	
Keine	5 % (18)
1–9	18 % (68)
10–25	24 % (90)
26–50	18 % (67)
51–75	11 % (40)
76–100	3 % (11)
> 100	2 % (6)
Unbekannt	16 % (60)
Sonstiges	1 % (3)
Keine Angabe	4 % (13)
*Haben Sie in Ihrer Einrichtung SOP zur perioperativen Versorgung von Patientinnen und Patienten?*	
Ja	76 % (247)
Nein	18 % (57)
Unbekannt	3 % (9)
Sonstiges (Freitext)	2 % (5)
Keine Angabe	2 % (6)
*Haben Sie in Ihrer Einrichtung eine SOP zur schmerztherapeutischen Versorgung von Majoramputationen?*	
Ja	33 % (83)
Nein	57 % (143)
Unbekannt	6 % (14)
Sonstiges (Freitext)	4 % (9)
Keine Angabe	1 % (3)
*Zu welchen Inhalten liegen SOP vor?*	
Narkoseplanung	82 % (75)
Prävention von Phantomschmerzen	63 % (57)
Postoperative Schmerzversorgung	92 % (84)
Sonstiges	3 % (3)

Die Umfrage zeigte hinsichtlich der Versorgungsprozesse, dass Patientinnen und Patienten zur Majoramputation nur sehr selten (lediglich von 3 % (*n* = 12) der Antwortenden angegeben) regelhaft mehr als 3 Tage vor der Operation in der Einrichtung der Anästhesiologie/Schmerzmedizin vorgestellt wurden. Von den Antwortenden gaben 48 % (*n* = 177) eine Vorstellung der Patientinnen und Patienten innerhalb von 3 Tagen vor der Operation an, 24 h vor Operation noch 36 % (*n* = 133) und am Operationstag 5 % (*n* = 17).

Maßnahmen zur Prävention von Phantomschmerz erfolgten laut 65 % (*n* = 238) der Antwortenden meist erst ab dem Operationstag selbst; lediglich 9 % (*n* = 31) der Antwortenden gaben an, dass am Vortag der Operation und zu 15 % „ein bis mehrere Tage“ vor der Amputation Maßnahmen erfolgten. Am häufigsten wurde angegeben, dass Anästhesistinnen und Anästhesisten (76 %) die schmerztherapeutische Planung übernahmen, wobei nach den Angaben mit 38 % das operative Fach und bei 34 % auch ein spezialisierter Schmerzdienst in die Versorgungsplanung eingeschlossen wurden (Mehrfachangaben möglich, Tab. 2, *Spalte A*).

**Table 2:** 

Frage	A) Angewandte Maßnahmen präoperative Schmerztherapie Häufigkeit % (*n*)	B) Als optimal empfundene Maßnahmen präoperative Schmerztherapie Häufigkeit % (*n*)
**Nichtopioidanalgetika** (Mehrfachauswahl)	69 % (239)	62 % (207)
*Bitte spezifizieren Sie Nichtopioidanalgetika*		
Saure antipyretische Antiphlogistika (z. B. Ibuprofen, Coxibe)	73 % (175)	78 % (156)
Nichtsaure Antipyretika (z. B. Paracetamol, Metamizol)	79 % (188)	80 % (159)
**Orale Opioide**	59 % (205)	53 % (179)
**Parenterale Opioide** (Mehrfachauswahl)	17 % (59)	18 % (59)
*Bitte spezifizieren Sie parenterale Opioide*		
Als Bolusgaben oder Kurzinfusionen	74 % (43)	51 % (30)
PCA, i.v. oder sublingual	59 % (34)	83 % (49)
**Regionalverfahren** (Mehrfachauswahl)	34 % (118)	74 % (249)
*Bitte spezifizieren Sie Regionalverfahren*		
Neuroaxiale Verfahren (z. B. Periduralkatheter)	55 % (64)	58 % (144)
Periphere Regionalanästhesie: Single-Shot	27 % (32)	17 % (43)
Periphere Regionalanästhesie: Katheterverfahren	85 % (99)	86 % (213)
**NMDA-Antagonisten** z. B. Ketamin	12 % (40)	24 % (81)
**Lidocain**, i.v.	4 % (15)	8 % (27)
**Gabapentin, Pregabalin**, präoperativ	36 % (126)	67 % (223)
**Antidepressiva** (z. B. Amitriptylin, Nortriptylin, Mirtazapin, Duloxetin)	21 % (72)	43 % (143)
**(Nicht-)medikamentöse Verfahren** (Mehrfachauswahl)	8 (27)	34 % (113)
*Bitte spezifizieren Sie (nicht-)medikamentöse Verfahren*		
Psychotherapie	44 % (12)	90 % (102)
Physiotherapie	96 % (26)	87 % (98)
Transkutane elektrische Nervenstimulation (TENS)	44 % (12)	50 % (57)
Wärme-/Kältetherapie	33 % (9)	29 % (33)
Spiegeltherapie	26 % (7)	46 % (52)
Prothesenversorgung	44 % (12)	50 % (56)
Sonstiges (Freitext)	0 % (0)	1 % (1)
**Sonstiges** (Freitext)	2 % (6)	3 % (9)
**Keine Therapie**	11 % (37)	1 % (3)
**Keine Angabe**	7 % (23)	4 % (13)

Für die intraoperative Versorgung wurden eine Allgemeinanästhesie (85 %, *n* = 316), eine Leitungs- oder Plexusanästhesie (63 %, *n* = 235) oder ein neuroaxiales Verfahren benannt (49 %, *n* = 182, Mehrfachangaben möglich). Die häufigsten Kombinationen waren neuroaxiale Verfahren mit peripheren Verfahren (28 %, *n* = 104), gefolgt von der Allgemeinanästhesie mit neuroaxialen und peripheren Verfahren (26 %, *n* = 95), der alleinigen Allgemeinanästhesie (13 %, *n* = 49) und der Allgemeinanästhesie mit neuroaxialen Verfahren (13 %, *n* = 48). In den Freitextkommentaren wurde wiederholt berichtet, dass eine Kombination der Allgemeinanästhesie mit Regionalverfahren und Stumpfkathetern durchgeführt würde. Des Weiteren wurde hier auf eine Anwendung von intravenösem Ketamin und Lidocain hingewiesen (Tab. 2).

## Vergleich der aktuellen perioperativen Praxis und einer als optimiert empfundenen perioperativen Versorgung

Auf Grundlage der Fallvignette wurde eine Analyse der aktuellen perioperativen Praxis und einer als optimiert empfundenen perioperativen Versorgung durchgeführt (Tab. 2, *Spalte B* zur „optimalen“ präoperativen Behandlung sowie Tab. 3, *Spalte B* zur „optimalen“ intra- und postoperativen Behandlung).

**Table 3:** 

Frage	A) Angewandte Maßnahmen zur Schmerztherapie ab der Operation Häufigkeit % (*n*)	B) Als optimal empfundene Maßnahmen zur Schmerztherapie ab der Operation Häufigkeit % (*n*)
**Nichtopioidanalgetika** (Mehrfachauswahl)	75 % (260)	71 % (234)
*Bitte spezifizieren Sie Nichtopioidanalgetika*		
Saure antipyretische Antiphlogistika (z. B. Ibuprofen, Coxibe)	74 % (189)	77 % (175)
Nichtsaure Antipyretika (z. B. Paracetamol, Metamizol)	88 % (225)	83 % (189)
**Orale Opioide**	53 % (183)	51 % (168)
Parenterale Opioide (Mehrfachauswahl)	72 % (250)	53 % (174)
*Bitte spezifizieren Sie parenterale Opioide*		
Als Bolusgaben oder Kurzinfusionen	72 % (176)	55 % (94)
PCA, i.v. oder sublingual	63 % (156)	81 % (139)
**Regionalverfahren** (Mehrfachauswahl)	84 % (289)	94 % (308)
*Bitte spezifizieren Sie Regionalverfahren*		
Neuroaxiale Verfahren (z. B. Periduralkatheter, Spinalanästhesie)	58 % (167)	61 % (188)
Regionale Nervenkatheter (z. B. Ischiadikuskatheter)	81 % (233)	85 % (260)
Regionale Single-Shot-Anästhesie (z. B. Femoralisblock)	43 % (123)	32 % (98)
Durch Operateur positionierte Nervenkatheter	35 % (101)	45 % (137)
Durch Operateur positionierte Stumpf- und Wundkatheter (nicht am Nerven)	17 % (50)	27 % (82)
Durch Operateur durchgeführte lokale Single-Shot-Verfahren (z. B. Wundinfiltration)	43 % (123)	35 % (106)
**NMDA-Antagonisten** z. B. Ketamin	45 % (156)	56 % (182)
**Lidocain**, i.v.	16 % (55)	23 % (75)
**Gabapentin, Pregabalin**	42 % (146)	66 % (215)
**Antidepressiva** (z. B. Amitriptylin, Nortriptylin, Mirtazapin, Duloxetin)	26 % (91)	45 % (148)
**Andere medikamentöse Verfahren** (Mehrfachauswahl)	7 % (24)	14 % (45)
*Bitte spezifizieren Sie andere medikamentöse Verfahren*		
Clonidin	79 % (19)	84 % (36)
Calcitonin	46 % (11)	28 % (12)
Cannabinoide	8 % (2)	35 % (15)
**Botulinumtoxin**	4 % (1)	16 % (7)
**(Nicht-)medikamentöse Verfahren** (Mehrfachauswahl)	10 % (33)	24 % (77)
*Bitte spezifizieren Sie (nicht-)medikamentöse Verfahren*		
Psychotherapie	33 % (11)	84 % (65)
Physiotherapie	82 % (27)	94 % (73)
Transkutane elektrische Nervenstimulation (TENS)	36 % (12)	61 % (47)
Wärme-/Kältetherapie	36 % (12)	36 % (28)
Spiegeltherapie	33 % (11)	70 % (54)
Prothesenversorgung	61 % (20)	79 % (61)
Sonstiges (Freitext)	0 % (0)	0 % (0)
**Sonstiges (Freitext)**	2 % (6)	2 % (6)
**Keine Therapie**	1 % (2)	0 % (0)
**Keine Angabe**	4 % (14)	2 % (7)

A) Präoperative Schmerztherapie: Welche Maßnahmen zur präoperativen Schmerztherapie wenden Sie regelhaft bei Majoramputationen wie in dem beschriebenen Fall in Ihrer Versorgungseinrichtung an (Mehrfachauswahl möglich)?

B) Optimale präoperative Schmerztherapie: Wenn Sie sich die Versorgung in der Zukunft vorstellen würden, welche Maßnahmen zur präoperativen Schmerztherapie entsprächen für Sie der optimalen Therapie bei Majoramputationen der unteren Extremität (Mehrfachauswahl möglich)?

**Figure 1:**
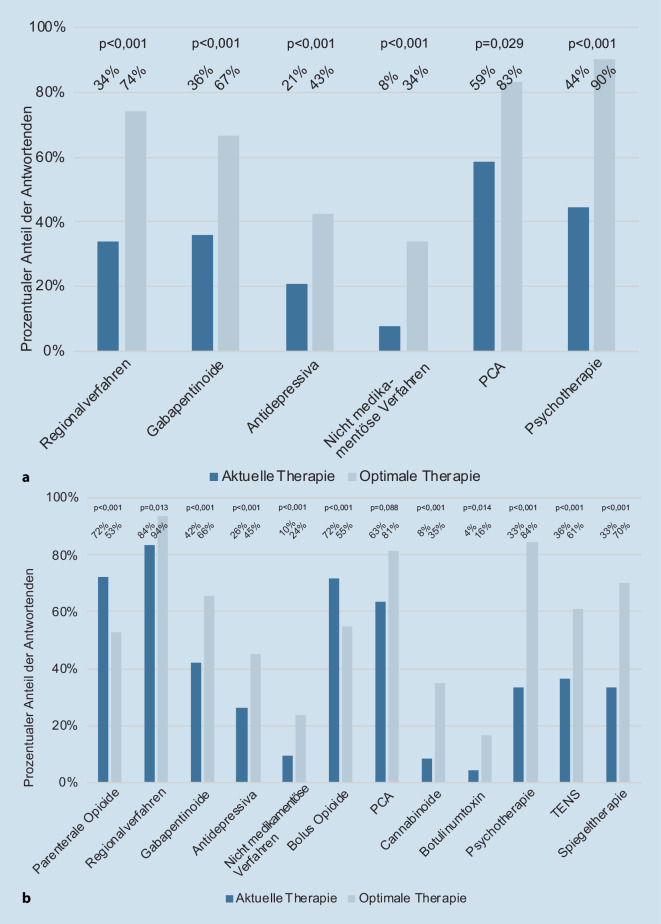

In den Angaben zu Vorstellungen einer optimalen Versorgung zeigte sich, dass die Antwortenden die Anlage präoperativer Regionalverfahren als früher indiziert ansahen, als dies in der gelebten Praxis erfolgte (Tab. 2). Auch zeigte sich hier die Einschätzung, Koanalgetika wie Gabapentinoide oder Antidepressiva häufiger nutzen zu wollen. Ebenso erschien der Einsatz einer präoperativen, patientenkontrollierten Analgesie mittels Opioids häufiger angedacht zu werden (Tab. 2, *Spalte B*). Unter den Angaben zu nichtmedikamentösen Verfahren wurde für eine optimale Versorgung neben Physiotherapie und physikalischen Maßnahmen wie Wärme‑/Kältetherapie oder transkutaner elektrischer Nervenstimulation (TENS) am häufigsten eine präoperative psychotherapeutische Betreuung (90 %, *n* = 102) benannt (Tab. 2). Ein Vergleich von Parametern mit relevanten Unterschieden in der Häufigkeit der Nennung zwischen der aktuellen Versorgung und einer als optimal empfundenen Versorgung findet sich in Abb. 1a, b.

Für die unmittelbare intra- und postoperative Versorgung zeigt Tab. 3, dass die Antwortenden ihren Vorstellungen einer optimalen Versorgung entsprechend weniger parenterale (Bolus‑)Opioide einsetzten, dafür häufiger Regionalverfahren, Koanalgetika sowie patientenkontrollierte Analgesie(PCA)-Verfahren präferierten. Von den Antwortenden, die „andere medikamentöse Verfahren“ wünschen, würden 35 % (*n* = 15) hier auch den Einsatz von Cannabinoiden sehen. Deutlich häufiger würden die Anästhesistinnen und Anästhesisten psychotherapeutische Ansätze sowie spezielle Verfahren, wie Spiegeltherapie oder transkutane elektrische Nervenstimulation (TENS), einsetzen. Dies deckte sich mit den Aussagen der Freitextkommentare, in denen ebenfalls häufig die Einbeziehung von Psychotherapie, Ergotherapie, Physiotherapie, multimodaler Schmerztherapie sowie Entspannungs- und Meditationsverfahren erwähnt wurde. Als spezielle Verfahren wurden im Freitext neben der bereits oben angeführten Spiegeltherapie auch die Akupunktur mitaufgeführt. Auch wurden hier Sozialdienste und etablierte Versorgungsstruktur für die Zeit nach der Entlassung aus der Akutbehandlung genannt.

## Barrieren gegenüber einer als optimal empfundenen Therapie in der aktuellen Praxis

In der Analyse von Barrieren gegenüber einer als optimal empfundenen Therapie standen unter den Nennungen v. a. organisatorische Faktoren im Vordergrund. Hierunter wurden sowohl ein limitierter zeitlicher Vorlauf bei Notfalleingriffen als auch eine späte Vorstellung dieser Patientinnen und Patienten in der Anästhesiologie in der Routineversorgung benannt. Patientenspezifika fanden ebenfalls häufig (65 %, *n* = 209) Erwähnung, wobei hier v. a. orale Antikoagulanzien (77 %, *n* = 159) und Infektionen (73 %, *n* = 151) eine limitierende Rolle zu spielen schienen. Eine fehlende Leitlinie mit spezifischen Inhalten wurde als allgemeiner Faktor von fast 70 % der Antwortenden genannt (Tab. 4). In den Freitextkommentaren wurde als häufigster Hinderungsgrund für eine optimale Schmerztherapie die fehlende Akzeptanz von Regionalverfahren der chirurgischen Kolleginnen und Kollegen angeführt. Als weitere Problemfelder wurden hier auch begrenzte Kapazitäten im Sinne eines Personal- und Zeitmangels sowie ungenügende Kommunikation zwischen den beteiligten Fachdisziplinen auf der einen und ungenügende Strukturen der schmerzmedizinischen Versorgung auf der anderen Seite erwähnt.

**Table 4:** 

Frage	Häufigkeit % (*n*)
*Aus Ihrer Beobachtung, welche der folgenden Faktoren führen zu einem Abweichen von der angedachten optimalen perioperativen Schmerztherapie (Mehrfachauswahl möglich)?*	
Patientenspezifische Faktoren	65 % (209)
Organisatorische Faktoren	78 % (253)
Allgemeine Faktoren	28 % (91)
Sonstiges (Freitext)	10 % (31)
Keine Angabe	4 % (13)
*Bitte spezifizieren Sie patientenspezifische Faktoren*	
Signifikante kardiopulmonale Begleiterkrankungen	52 % (108)
Einnahme oraler Antikoagulation	77 % (159)
Infektionen	73 % (151)
*Bitte spezifizieren Sie organisatorische Faktoren*	
Notfalloperation (N0–N4)	58 % (144)
Operation außerhalb der Regelarbeitszeit	43 % (107)
Späte Patientenvorstellung in der Anästhesie	82 % (204)
*Bitte spezifizieren Sie allgemeine Faktoren*	
Unklare Evidenzlage	57 % (48)
Fehlende Leitlinie	69 % (59)
Off Label Use der Medikamente	33 % (28)

*N0–N4* Klassifikation der Dringlichkeit von Operationen nach Bauer et al. [1]

## Diskussion

Insgesamt zeigte sich in dieser Studie aus anästhesiologisch-fachärztlicher Sicht ein heterogenes Bild des Schmerzmanagements in der aktuellen perioperativen Versorgung von Patientinnen und Patienten mit Majoramputation. Die Ergebnisse lassen vermuten, dass ein nichtgedeckter Bedarf an spezifischen perioperativen schmerztherapeutischen Behandlungskonzepten besteht. Die im Frühjahr 2022 erschienene Leitlinie gibt hier eine Übersicht über aktuell empfohlene Methoden der Phantomschmerzprävention [31]. Solche evidenzbasierte Maßnahmen werden von zahlreichen Kliniken in lokale Handlungsanweisungen im Sinne von „standard operating procedures“ (SOP) überführt, da diese eine der zentralen Säulen der Steuerung des Behandlungsprozesses und der Komplikationsvermeidung in der Anästhesie und Intensivmedizin darstellen [6]. Auch wenn ein Großteil der Antwortenden in der vorliegenden Studie angab, dass solche SOP etabliert seien, fehlten jedoch meist Aspekte zum Schmerzmanagement von Amputationen. Hier gibt es also deutliches Potenzial zur Weiterentwicklung.

In der vorliegenden Umfrage konnte für zahlreiche Aspekte ein relevanter Unterschied zwischen der aktuellen Praxis der Versorgung und einer aus Sicht der Antwortenden als optimiert attribuierten perioperativen Versorgung von Patientinnen und Patienten mit Majoramputation festgestellt werden. So wurde deutlich, dass laut den Umfrageteilnehmerinnen und -teilnehmern eine Schmerztherapie den Schwerpunkt weniger auf Opioide, sondern mehr auf Regionalverfahren legen sollte. Dies entspricht der aktuellen Akutschmerzleitlinie, in der periphere Nervenblockaden, Periduralanästhesie oder – wenn erstere der beiden Optionen nicht möglich – nervennahe Katheter durch den Operateur angewandt werden sollten (GoR B) [31]. Laut unserer Umfrage könnten eingesetzte Opioide möglicherweise günstiger im Rahmen patientenkontrollierter Analgesieverfahren (PCA) Anwendung finden. Auch dies deckt sich mit den Empfehlungen der Leitlinie, laut der für die Akutschmerztherapie eine intravenöse patientenkontrollierte Analgesie mit einem Opioid genutzt werden sollte (GoR B) [31]. Solche Verfahren führen auf der einen Seite zu mehr Patientenzufriedenheit und Autonomie, auf der anderen Seite wurden höhere Opioiddosierungen beobachtet und damit vermutlich die potenzielle analgetische Unterversorgung reduziert [19]. In Bezug auf die Wirksamkeit von regionalanästhesiologischen Verfahren in der Phantomschmerzprävention ist die bisherige Literaturlage uneinheitlich. In einer Metaanalyse konnte kein unmittelbarer Effekt von Perineuralkathetern auf postoperative Schmerzen, Mortalität und die Entstehung von Phantom- oder Stumpfschmerzen nach Amputationen gezeigt werden, jedoch konnte der Opioidkonsum etwa halbiert werden [2]. Der Einsatz von Lokalanästhetika und deren Kombination mit Opioiden scheint sinnvoll, um Opioide zu sparen und damit deren Nebenwirkungen wie Sedierung, Atemdepression, Übelkeit, Obstipationen und Suchtentstehung zu vermeiden [26].

Neben Lokalanästhetika und Opioiden wurde auch der Einsatz von NMDA-Antagonisten, insbesondere von Ketamin, diskutiert. Hier gab knapp die Hälfte der Antwortenden an, Ketamin tatsächlich einzusetzen, während sich ein etwas größerer Anteil dies in einer optimalen Behandlung wünschen würde. Dieser verhältnismäßig zurückhaltende Einsatz steht im Widerspruch zur aktuellen Leitlinie, in der Ketamin bei Amputationen für die Akutschmerztherapie eingesetzt werden soll (GoR A) und auch zur Phantomschmerzprophylaxe beitragen könnte (GoR 0) [31].

Ein anderer Aspekt ist die von den Antwortenden angeführte häufigere perioperative Gabe von Gabapentinoiden, was Gegenstand der aktuellen Diskussion für die Phantomschmerzprävention ist. Auf der einen Seite konnten Nikolajsen et al. in einem RCT mit 41 Patientinnen und Patienten, die postoperativ für 30 Tage Gabapentin erhielten, keinen Unterschied in der Inzidenz oder Intensität von Postamputationsschmerzen feststellen [20]. Auf der anderen Seite wurden in einem aktuelleren RCT mit 45 pädiatrischen Patientinnen und Patienten, die eine Amputation aufgrund maligner Knochentumoren in Allgemeinanästhesie erhielten und dafür 4 Tage präoperativ Gabapentin oral verabreicht bekamen, eine signifikant geringere Schmerzintensität und eine geringere Rate von Phantomschmerzen in der 60 Tage-Follow-up-Visite beobachtet [30]. Die Ergebnisse einer Metaanalyse zur Nutzung von Gabapentinoiden für die postoperative Akutschmerzbehandlung ergaben zwar statistisch signifikante, aber klinisch nichtrelevante Unterschiede für postoperative Schmerzen, weshalb der routinemäßige Einsatz von den Autoren der Analyse nicht empfohlen wird [29]. Auch laut der aktuellen Akutschmerzleitlinie sollte Gabapentin perioperativ nicht zur Prävention von Phantomschmerz eingesetzt werden (GoR B) [31]. Unabhängig von der Prävention sind Gabapentinoide jedoch ein fester Bestandteil der Therapie von bereits etablierten neuropathischen Schmerzen [23].

In unserer Umfrage zeigt sich, dass nichtmedikamentöse Verfahren nur recht selten in der frühen perioperativen Phase im Rahmen von Majoramputationen angewandt werden (10 %, *n* = 33), in der optimierten Behandlung jedoch häufiger Anwendung fänden (24 %, *n* = 77). Unter diesen Verfahren war laut Umfrage die Physiotherapie sowohl in der tatsächlichen Versorgung (82 %, *n* = 27), als auch in einer als optimal angenommenen Versorgung (95 %, *n* = 73) führend bei den Angaben. Physiotherapeutische Maßnahmen fokussieren sich in der klinischen Praxis auf chirurgischen Stationen oft v. a. auf die frühzeitige Mobilisation der Patientinnen und Patienten sowie die Vorbeugung von spezifischen perioperativen Komplikationen [3].

Die Anwendung von TENS erfolgt in der aktuellen Praxis den Angaben zufolge selten perioperativ (36 %, *n* = 12), eine Steigerung wäre jedoch den Antworten entsprechend anscheinend wünschenswert (61 %, *n* = 47). Die geringe Nutzung von TENS ist auch in der Literatur beschrieben [3]. Es liegen zur Anwendung von TENS in der Phantomschmerzprävention nur anekdotische Einzelfallberichte und Fallserien insbesondere aus den 1980er-Jahren vor, sodass keine sicheren Therapieempfehlungen abgeleitet werden können [14]. So findet sich in der aktuellen Leitlinie lediglich die Empfehlung, dass TENS nach Amputation für die Akutschmerztherapie angewendet werden kann (GoR 0) [31].

Psychotherapeutische Ansätze könnten u. a. bezüglich realistischer Therapieziele, der Annahme und Akzeptanz der Veränderungen des Körperschemas und ggf. vor dem Hintergrund komorbider, depressiver, emotionaler Störungen, die moderat bis stark mit einer Phantomschmerzentwicklung assoziiert sind, relevant sein [18]. Auch psychologische Faktoren, wie Katastrophisierung oder Hoffnung, konnten hier eine starke Assoziation zeigen und könnten somit hilfreiche Therapieansätze darstellen [18, 22]. Jedoch scheint die Rolle von psychologischen Faktoren in der Entstehung von Phantomschmerzen geringer zu sein als bei anderen Schmerzerkrankungen [10]. So wurde beschrieben, dass affektive Störungen wie Depressionen eher den Verlauf und die Schwere von Phantomschmerzen modellieren als einen direkten Einfluss auf die Entstehung von diesen haben [7]. Ein psychotherapeutischer Erstkontakt in der perioperativen Phase könnte jedoch auch für diese Fachdisziplin eine frühzeitigere transsektorale Anbindung von Patientinnen und Patienten in entsprechende Nachversorgungsstrukturen bahnen [15, 16]. Neben einer perioperativen Psychotherapie wird in der Literatur auch die Anwendung von präemptiver Psychoedukation diskutiert. Diese könnte über Angstreduktion das Komplikationsrisiko senken und somit über eine kürzere Krankenhausverweildauer und geringere Analgetikanutzung u. a. auch Kosten sparen [13].

Die Barriereanalyse zeigte aus Sicht der Befragten zahlreiche Hinderungsgründe auf, die einer optimierten Versorgung entgegenstehen. Als größtes Problemfeld wurden organisatorische Faktoren aufgeführt, wobei insbesondere eine späte Vorstellung der Patientinnen und Patienten in der Anästhesie im Fokus stand. Eine solche späte Vorstellung schränkt eine Risikoevaluation und die Möglichkeiten präventiver Therapieansätze deutlich ein [7, 27]. Aus den Freitextkommentaren wurde des Weiteren deutlich, dass es auch in der interdisziplinären Zusammenarbeit insbesondere mit den Kolleginnen und Kollegen der chirurgischen Disziplinen Optimierungspotenzial geben könnte. Als Hauptgrund für eine Abweichung von der optimalen Therapie in der klinischen Realität wurde in den Kommentaren die Ablehnung von Regionalverfahren durch chirurgische Kolleginnen und Kollegen beschrieben. Andere häufig genannten Probleme waren Zeitdruck bei der Arbeit und eingeschränkte Personalressourcen.

Beides stellt die Umsetzung von personalintensiven, präventiven Maßnahmen vor eine große Herausforderung. Um solche Präventionskonzepte umsetzen zu können, sind somit neben der Erforschung von deren Wirksamkeit auch ein Umdenken bezüglich der Ausrichtung medizinischer Versorgungsangebote und Strategien sowie eine entsprechende Anpassung und Ausgestaltung der hierzu erforderlichen Versorgungsstrukturen durch die Gesundheitspolitik und die Kostenträger notwendig. Um eine ausreichende Datengrundlage dafür zur Verfügung zu haben, wäre es sinnvoll, ein Amputationsregister mit Routinedaten der Patientinnen und Patienten zu erschaffen.

## Limitationen

Grundsätzlich ließ sich die Rücklaufquote dieser Umfrage nicht präzise ermitteln, da aufgrund datenschutzrechtlicher Auflagen eine Erhebung von personenbezogenen Daten – insbesondere in der Grundgesamtheit von Adressaten – nicht möglich war und diese mit der vorliegenden Stichprobe nicht verglichen werden konnten. Dies schränkt Aussagen zur Repräsentativität ein. Eine weitere Limitation liegt darin, dass nur Anästhesistinnen und Anästhesisten und keine weiteren Disziplinen zu der Umfrage eingeladen wurden. An dieser Umfrage haben zudem hauptsächlich mit Ärztinnen und Ärzten in Leitungsposition in größeren Krankenhäusern (Schwerpunkt‑/Zentralversorgung, Maximalversorgung und Universitätsklinik), die als Traumazentren zertifiziert sind, teilgenommen. So könnten die hier zusammengestellten Antworten möglicherweise die Überzeugungen einer Gruppe von Ärztinnen und Ärzten mit besonderem Interesse an der Behandlung von Patientinnen und Patienten mit Amputationen widerspiegeln.

Des Weiteren wurde in der Umfrage generell zu Amputationen gefragt, weshalb Unterschiede im Management von Amputationen verschiedener Genese (z. B. traumatisch, angiologisch) nicht abgebildet werden können. Jedoch spielen traumatische Amputationen in Deutschland und international mit ca. 11–22 % an der Gesamtzahl der Amputationen eine untergeordnete Rolle [17, 28].

Für das Erreichen von Teilnehmern war insbesondere der Einfluss von E‑Mail-Filtern und ähnlichen Schutzsystemen nicht vorherzusehen. Laut persönlicher Mitteilung hat jedoch eine ganze Reihe von Kolleginnen und Kollegen die Einladungen zur Umfrage zunächst nicht erhalten. Ein weiteres Problem bei Umfragen dieser Art ist die häufige Diskrepanz zwischen Antworten und der Realität. Zur Objektivierung der Antworten könnte die angegebene Anzahl an Amputationen pro Klinik herangezogen werden. Die Einschätzung der Anzahl der Operationen in den jeweiligen Versorgungseinrichtungen in unserer Umfrage deckte sich recht genau mit populationsbasierten Daten mittels DRG-Statistik (diagnosebezogene Fallgruppen) von 2005 bis 2015 zu Amputationen der unteren Extremität in Deutschland: Die Mehrzahl der Antworten in unserer Studie lag innerhalb des Interquartilabstands der medianen jährlichen Fallzahlen für Amputationen der unteren Extremität pro Klinikum (Median 25, IQR 11–56 [2005]; Median 33, IQR 10–75 [2015]) [25].

## Schlussfolgerung bzw. „Fazit für die Praxis“

In der perioperativen Versorgung von Patientinnen und Patienten mit Majoramputation werden regionalanästhesiologische Verfahren zu 84 % eingesetzt, könnten jedoch entsprechend der aktualisierten Leitlinienempfehlung noch häufiger zum Einsatz kommen. Insbesondere Infektionen, Antikoagulation und eine späte Vorstellung der Patientinnen und Patienten in der Anästhesie werden als aktuelle relevante Barrieren benannt. Eine frühere anästhesiologische Einbindung zur Identifikation von Risikokonstellationen und deren frühzeitige Behandlung könnten die perioperative Versorgung optimieren.
